# High-flow nasal cannula oxygen therapy is superior to conventional oxygen therapy but not to noninvasive mechanical ventilation on intubation rate: a systematic review and meta-analysis

**DOI:** 10.1186/s13054-017-1760-8

**Published:** 2017-07-12

**Authors:** Huiying Zhao, Huixia Wang, Feng Sun, Shan Lyu, Youzhong An

**Affiliations:** 1 Department of Critical Care Medicine, Peking University People’s Hospital, No. 11 Xizhimen South Street, Xicheng District, Beijing, 100044 China; 2 Department of Epidemiology and Biostatistics, School of Public Health, Peking University Health Science Center, Beijing, 100038 China

**Keywords:** High-flow nasal cannula oxygen (HFNC), Conventional oxygen therapy (COT), Noninvasive mechanical ventilation (NIV), Intubation, Mortality

## Abstract

**Background:**

High-flow nasal cannula oxygen (HFNC) is a relatively new therapy used in adults with respiratory failure. Whether it is superior to conventional oxygen therapy (COT) or to noninvasive mechanical ventilation (NIV) remains unclear. The aim of the present study was to investigate whether HFNC was superior to either COT or NIV in adult acute respiratory failure patients.

**Methods:**

A review of the literature was conducted from the electronic databases from inception up to 20 October 2016. Only randomized clinical trials comparing HFNC with COT or HFNC with NIV were included. The intubation rate was the primary outcome; secondary outcomes included the mechanical ventilation rate, the rate of escalation of respiratory support and mortality.

**Results:**

Eleven studies that enrolled 3459 patients (HFNC, *n* = 1681) were included. There were eight studies comparing HFNC with COT, two comparing HFNC with NIV, and one comparing all three. HFNC was associated with a significant reduction in intubation rate (OR 0.52, 95% CI 0.34 to 0.79, *P* = 0.002), mechanical ventilation rate (OR 0.56, 95% CI 0.33 to 0.97, *P* = 0.04) and the rate of escalation of respiratory support (OR 0.45, 95% CI 0.31 to 0.67, *P* < 0.0001) when compared to COT. There was no difference in mortality between HFNC and COT utilization (OR 1.01, 95% CI 0.67 to 1.53, *P* = 0.96). When HFNC was compared to NIV, there was no difference in the intubation rate (OR 0.96; 95% CI 0.66 to 1.39, *P* = 0.84), the rate of escalation of respiratory support (OR 1.00, 95% CI 0.77 to 1.28, *P* = 0.97) or mortality (OR 0.85, 95% CI 0.43 to 1.68, *P* = 0.65).

**Conclusions:**

Compared to COT, HFNC reduced the rate of intubation, mechanical ventilation and the escalation of respiratory support. When compared to NIV, HFNC showed no better outcomes. Large-scale randomized controlled trials are necessary to prove our findings.

**Trial registration:**

PROSPERO International prospective register of systematic reviews on May 25, 2016 registration no. CRD42016039581.

**Electronic supplementary material:**

The online version of this article (doi:10.1186/s13054-017-1760-8) contains supplementary material, which is available to authorized users.

## Background

High-flow nasal cannula oxygen (HFNC) is a relatively new and increasingly used therapy in adults with acute respiratory failure [1]. HFNC can deliver heated humidified oxygen through nasal prongs and provide much higher and more predictable rates of gas flow (maximum flow of 60 L/min) and fraction of inspired oxygen (FiO_2_) (up to 1.0) [2–4].

HFNC has several advantages when compared to conventional oxygen therapy (COT): (1) the high-flow rates match the patient’s inspiratory flow rates, which creates a positive pressure effect [5, 6] and reduces the anatomic dead space [7]; (2) HFNC can deliver a predictable and constant FiO_2_ [8]; (3) HFNC can increase the partial arterial pressure of oxygen (PaO_2_)/FIO_2_ ratio, which reduces the entrainment of room air and the dilution of oxygen [1, 2]; (4) the heated and humidified gas that is inhaled can improve mucociliary motion and sputum clearance [3, 9]; and (5) there is reduced upper airway resistance, reduced work of breathing [10] and improvement in thoraco-abdominal synchrony [11, 12]. Based on the above advantages, several studies found that HFNC could improve comfort level [13–15], increase oxygenation [12, 14–17] and decrease the dyspnoea score in adult patients [3, 12, 14, 17]. Nevertheless, there has been no clear consensus on treatment outcomes (such as intubation rate, escalated respiratory support rate and mortality) [13, 18–22].

Compared to HFNC, noninvasive mechanical ventilation (NIV) can create a much higher gas flow rate and positive airway pressure but is not comfortable and has many complications [23, 24]. Recently, well-designed randomized controlled trials (RCTs) have not differentiated the effect of HFNC and NIV on intubation rates and mortality [18, 25]. To decrease complexity, a recently published meta-analysis showed no significant difference in mortality or intubation rates when HFNC was compared to COT and NIV [26]. The conclusion of this study might be misleading: first, COT and NIV are very different in terms of the mechanism of action and clinical application; second, the combination of COT and NIV not only increased patient heterogeneity but also increased the power of statistical bias. A separate comparison of HFNC with COT and NIV should result in more reasonable conclusions. Maitra et al. included only six RCTs in their meta-analysis that compared only the prognosis of higher respiratory support, and found no significant difference between HFNC and either standard oxygen therapy or NIV [27]. The small number of studies included in the meta-analysis makes its application limited.

Therefore, we conducted a meta-analysis to investigate the effect of HFNC on the rates of intubation, mechanical ventilation, the escalation of respiratory support and its effect on mortality versus COT or NIV in adult patients with respiratory failure.

## Methods

We conducted a systematic review in accordance with the methods recommended in the Preferred Reporting Items for Systematic Reviews and Meta-Analyses (PRISMA) guidelines [28].

### Search strategy

Using electronic and manual searching, a literature review was performed from the electronic databases inception up to 20 October 2016 in the Cochrane Library, Embase and Ovid Medline without language restrictions. The following search terms were used: ‘hfnc’, ‘hfnp’, ‘hhfnox’, ‘hfno’, ‘(high flow) adj5 nasal’, ‘(high flow) adj5 oxygen’, ‘high-flow adj5 nasal’ and ‘nasal adj5 (high flow)’. The search strategy is in the Appendix (Additional file 1). In addition, we manually searched clinical trials.gov and the bibliographies of randomized controlled trials, meta-analyses, and systematic reviews to identify other potentially relevant studies. The references of all included articles were also checked manually to identify additional eligible studies. The literature review was conducted independently by two authors (HYZ and HXW). Disparities in the literature review were resolved by a consensus of all authors.

### Inclusion and exclusion criteria

The following criteria were used for inclusion in our meta-analysis:

1. Type of study: randomized controlled trials;
2. Population: adult patients with respiratory failure who received oxygen therapy;
3. Intervention: HFNC treatment compared either COT or NIV;
4. Predefined outcomes: intubation, mechanical ventilation (includes noninvasive mechanical ventilation and invasive mechanical ventilation), escalation of respiratory support (HFNC, NIV or intubation) and mortality.

If there was more than one eligible trial from one team, the study with the most recent publication date was used in the analysis.

Exclusion criteria: the types of articles excluded from the analysis were reviews, retrospective studies, observational studies, case reports, animal studies, studies conducted on children, studies examining only psychological mechanisms, unrelated studies (e.g., HFNC not used in patients), duplicate reports, studies involving repeated experiments (commentary articles on specific studies or secondary analyses of experimental data), studies not in either the English or the Chinese language and non-randomized trials.

The primary outcome of our study was to investigate whether HFNC versus COT or HFNC versus NIV resulted in a similar intubation rate. The secondary outcomes were whether HFNC versus COT had similar rates of mechanical ventilation and rates of escalation of respiratory support and mortality, and whether HFNC versus NIV had similar rates of escalation of respiratory support and mortality.

### Data extraction

Two reviewers (HYZ and HXW) extracted the data independently using a predefined data extraction form. Disagreements were resolved through discussion or consensus with a third reviewer (FS). The data extracted included the study ID (together with the first author’s name and publication year), country, study design, setting, control therapy, duration of therapy, primary and secondary outcomes and clinical outcomes. We also checked the supplementary files and contacted the authors for more detailed information if necessary.

### Quality assessment and publication bias

Two independent reviewers assessed the methodological quality of the included trials using the Cochrane Collaboration Risk of Bias tool [29] within the RevMan5.3 software, which considers seven different domains: adequacy of sequence generation; allocation sequence concealment; blinding of participants and caregivers; blinding for outcome assessment; incomplete outcome data; selective outcome reporting; and the presence of other potential sources of bias not accounted for in the other six domains (the current research primarily refers to receiver sponsorship or oxygen therapy devices from Fisher and Paykel Healthcare or from other organizations). Based on the method of the trials, each was graded as “yes”, “no” or “unclear”, to reflect a high, low risk or uncertain risk of bias, respectively. Two reviewers (HYZ and HXW) made judgments independently. In cases of disagreement, resolution was first attempted by discussion and then by consulting a third author (FS) for arbitration.

Funnel plots were used to assess the possibility of publication bias and were implemented in RevMan 5.3 software. The Egger regression test was used to measure funnel plot asymmetry [30] and was implemented using Stata 12.0 (StataCorp LP, TX, USA).

### Grading the quality of the evidence

We used the methodology of the Grades of Recommendation, Assessment, Development and Evaluation (GRADE) Working Group to assess the overall quality of the evidence for the primary and secondary outcomes in the following domains: risk of bias, inconsistency, indirection, imprecision and publication bias. These were classified as very low, low, medium and high [31].

### Statistical analysis

Statistical analyses were performed using RevMan 5.3 software (Cochrane Library, London, UK). We combined data from all trials to estimate the pooled odds ratio (OR) with 95% confidence intervals (CIs) for the rates of intubation, mechanical ventilation, escalation of respiratory support and mortality. Pooled ORs with 95% CI were estimated by the Mantel-Haenszel method. ORs were undefined and excluded for studies with no event in either arm. The analysis was performed using a random effects model. Heterogeneity was tested using a weighted Mantel-Haenszel *χ* ^*2*^ test and was quantified using the *I* ^*2*^ statistic as implemented in RevMan *I* ^*2*^ values, which describes the percentage of the variability in effect and computes estimates that are due to heterogeneity rather than sampling error. *I* ^*2*^ values of 25–50% indicated low, 50–75% indicated moderate, and >75% indicated high heterogeneity. A value >50% may be considered substantial heterogeneity [32]. A *P* value of 0.1 was used to denote the statistical significance of heterogeneity. Differences between subgroups were analysed using the test of subgroup differences described by Deeks et al., and the results were expressed using the *P* values. *P* < 0.05 was considered statistically significant.

### Subgroup analysis

We utilized subgroup analysis to assess possible influences of the oxygen therapy system on clinical outcomes, which allowed us to explore the possible causes of the heterogeneity. In the comparison of HFNC to COT, we first explored whether there was a different treatment effect of the oxygen therapy system in patients with post-extubation acute respiration failure (ARF) and patients with ARF that occurred for other reasons. Second, we assessed the effect of trials that allowed COT to escalate to HFNC versus those studies that did not. Moreover, we also compared the results of RCTs with patients from single-centre studies versus patients from multi-centre studies.

## Results

### Study identification and selection

**Fig. 1:**
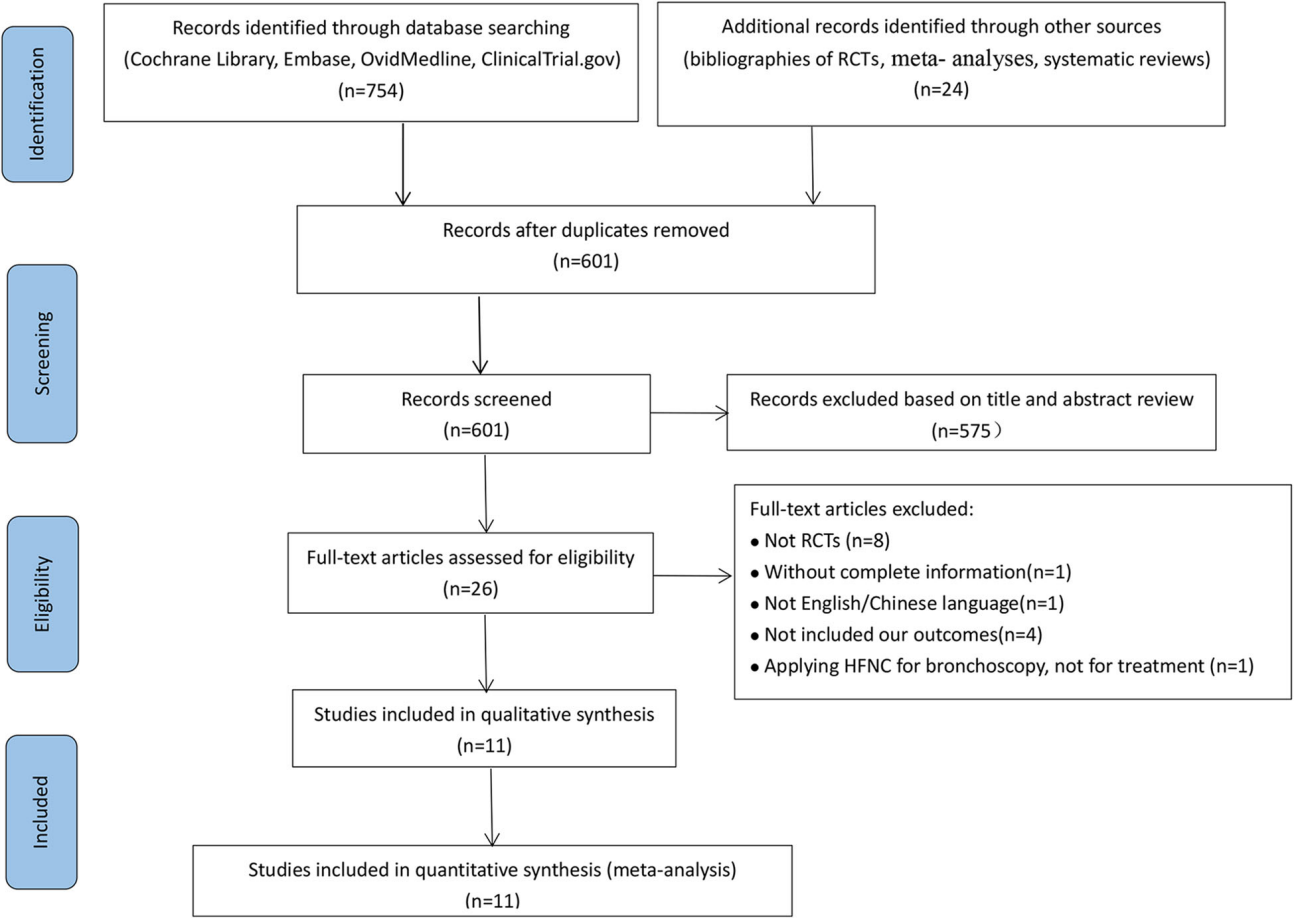
Selection of studies included in this meta-analysis. *RCT* randomized controlled trial, *HNFC* high-flow nasal cannula oxygen

The initial search of the database revealed 754 articles, and other sources revealed 24 articles. After the removal of duplicates, there were 601 articles that were screened based on their titles and abstracts to identify potentially eligible trials. The full texts of 26 articles were assessed, and 15 studies available in full text were excluded: 8 were not RCTs [10, 15, 16, 33–37]; 1 was not in the English or Chinese language [38]; 4 did not include the outcomes of our meta-analysis [39–42]; and 1 applied HFNC for bronchoscopy but not for treatment [43] (Additional file 2). In total, 10 RCTs (11 studies, 1 study compared all three groups) [13, 18–22, 25, 44–47] were eligible and were included in this meta-analysis, which ultimately included 3459 subjects. A PRISMA flow diagram of the selection of studies is shown in Fig. 1.

The characteristics of individual studies included in this meta-analysis are presented in Table 1. There was one trial published over two articles: HFNC versus COT [19] and HFNC versus NIV [25]. Eight studies [13, 19–22, 44–46] compared HFNC with COT, and two studies [25, 47] compared HFNC with NIV, whereas another study [18] compared HFNC with both. Two studies [13, 20] were conducted in emergency departments (EDs), and others were conducted in intensive care units (ICUs). Eight were multi-centre studies. There were two studies performed in Australia [13, 44], three in France [18, 45, 47], three in New Zealand [20, 22, 46], two in Spain [19, 25] and one in Italy [21].

**Table 1:** Main characteristics of the 11 studies included in the meta-analysis

Study	Country	Setting	Study design	Patients	Control	Duration (h)	Primary outcomes	Secondary outcomes		
							Intubation	Mechanical ventilation	Escalation	Mortality
Bell N, 2015^a^ [13]	Australia	ED	Multi-centre	Acute undifferentiated shortness of breath	FM/nasal prongs	2 h	Yes	Yes	Yes	No
Corley A, 2015^a^ [44]	Australia	ICU	Multi-centre	Post-extubation after cardiac surgery with BMI ≥30 kg/m^2^	FM/nasal cannula	24 h	Yes	Yes	Yes	No
Frat JP, 2015 [18]	France	ICU	Multi-centre	AHRF (without hypercapnia)	FM/NIV	48 h	Yes/yes	Yes/-	Yes/yes	Yes/yes
Hernandez G^1^, 2016 [19]	Spain	ICU	Multi-centre	Post-extubation RF in low risk for reintubation	FM/nasal cannula	24 h	Yes	Yes	Yes	Yes
Jones PG, 2015 [20]	New Zealand	ED	Single-centre	Hypoxia and tachypnea	FM/nasal prongs	3 h	Yes	Yes	Yes	Yes
Lemiale V, 2015 [45]										
	France	ICU	Multi-centre	Immunocompromised patients with AHRF	FM	2 h	Yes	Yes	Yes	No
Maggiore SM, 2014 [21]	Italy	ICU	Multi-centre	Post-extubation ARF	FM	48 h	Yes	Yes	Yes	Yes
Parke R, 2013^a^ [22]	New Zealand	CVICU	Single-centre	Post-extubation after cardiac surgery	FM/or nasal prongs	24 h	Yes	Yes	Yes	Yes
Parke R, 2011 [46]	New Zealand	CVICU	Single-centre	Mild to moderate AHRF	FM	24 h	_	Yes	Yes	No
Stephan F, 2015 [47]	France	CTVS ICU	Multi-centre	ARF after cardiothoracic surgery	NIV	Period of ICU stay	Yes	_	Yes	Yes
Hernandez G^2^, 2016 [25]	Spain	ICU	Multi-centre	Post-extubation RF in high risk for reintubation	NIV	24 h	Yes	_	Yes	Yes

*CTVS* cardiothoracic and vascular surgery, *ICU* intensive care units, *CVICU* cardiothoracic and vascular ICU, *COT* conventional oxygen therapy, *NIV* noninvasive mechanical ventilation, *ED* emergency department, *BMI* body mass index, *AHRF* acute hypoxaemic respiratory failure, *RF* respiratory failure, *FM* face mask

^a^In these studies, the group of patients who received COT could be escalated to HFNC if necessary, whereas the other patients were not escalated to HFNC

Hernandez G^1^ [19] 2016 and Hernandez G^2^ [25] 2016 were two articles from the same trial

**Fig. 2:**
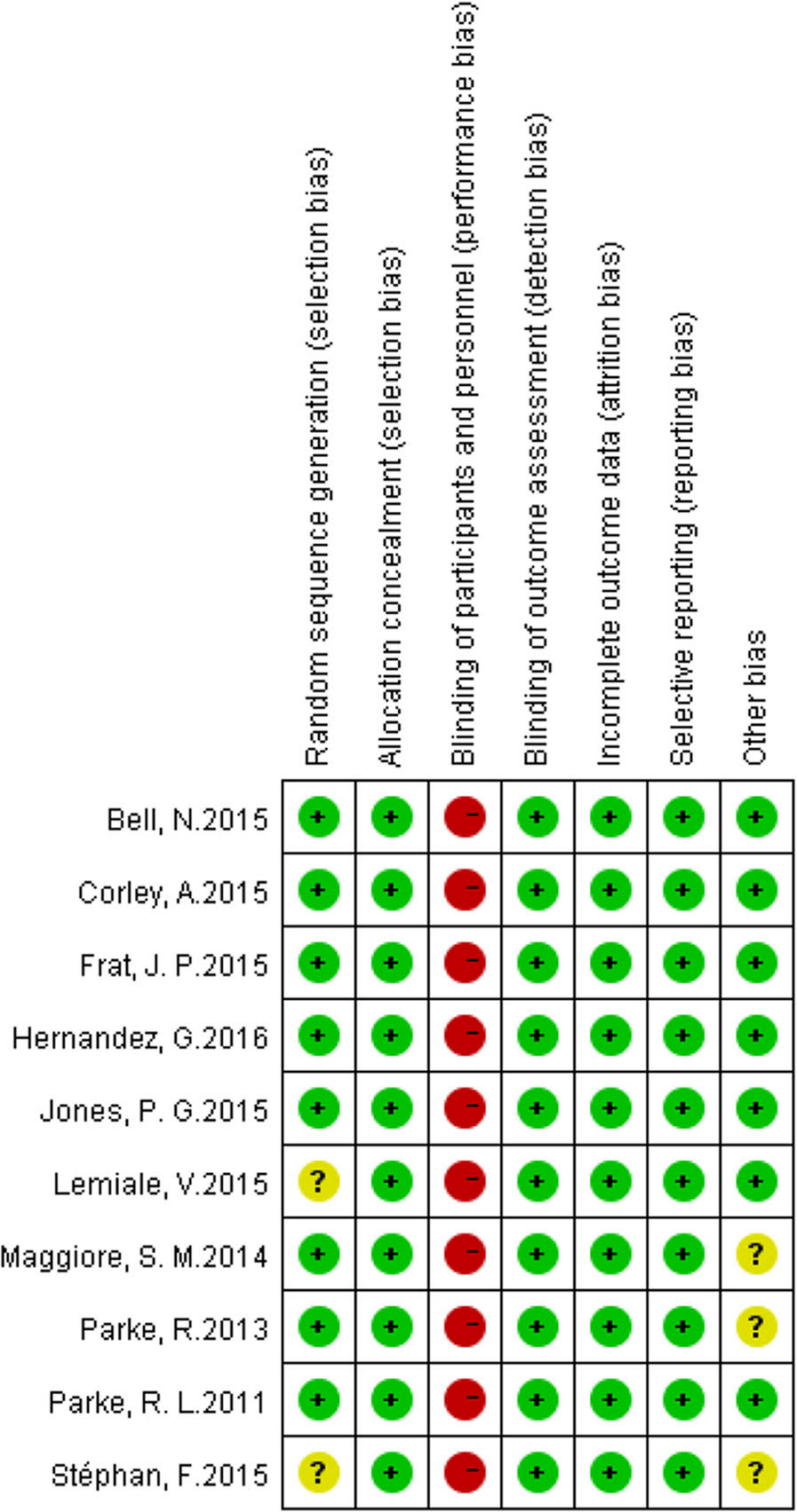
Methodological quality of trials using the Cochrane risk of bias tool. Symbols show low risk of bias (*+*), unclear risk of bias (?) or high risk of bias (-)

**Fig. 3:**
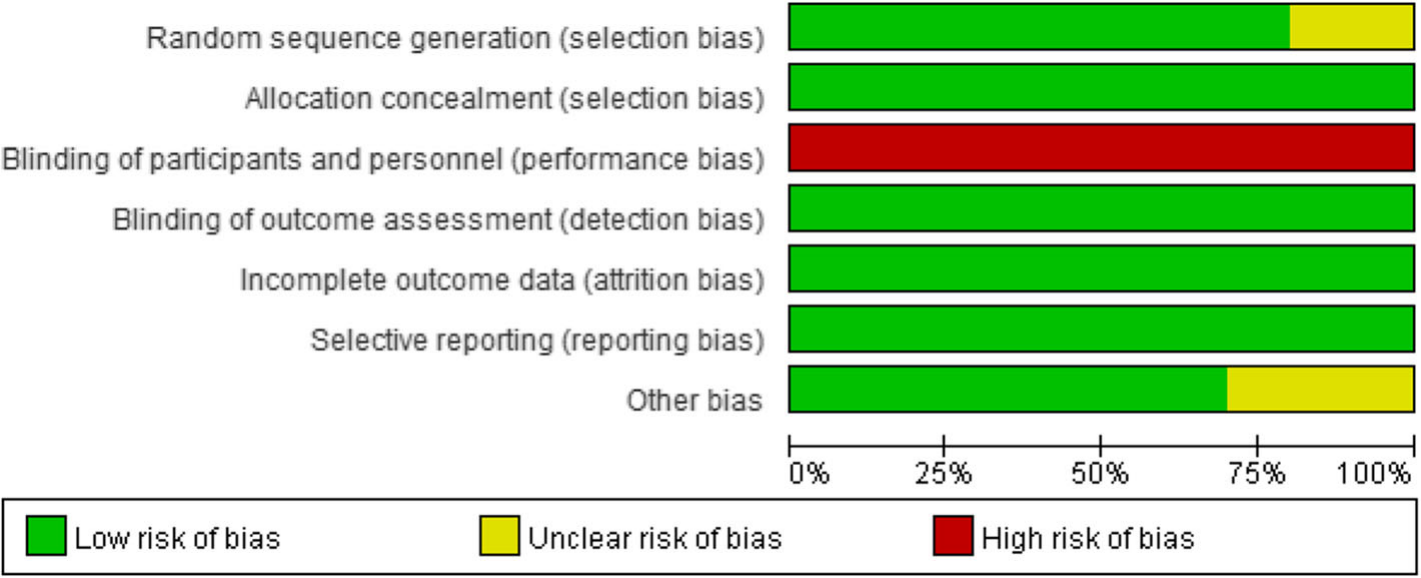
Overall risk of bias using the Cochrane risk of bias tool

For all RCTs in this meta-analysis, most of the domains were evaluated as having low risk of bias (allocation sequence concealment, blinding for outcome assessment, incomplete outcome data and selective outcome reporting of domains). Most notably, blinding of participants and personnel was not possible in these trials because of the dramatic differences between HFNC, COT and NIV; therefore, performance bias was considered to be a high risk in all the studies. Other types of bias are herein referred to as commercial interference. Seven of the articles [13, 18–20, 44–46] stated that the trial design and data analysis was independent of commercial interference; nevertheless, another three studies [21, 22, 47] did not make this clarification. In addition, randomization of sequence generation was unclear in two studies [45, 47] because they did not describe the specific methods of randomization. The risk of summary bias in individual studies is shown in Fig. 2, Fig. 3 and Additional file 3.

**Fig. 4:**
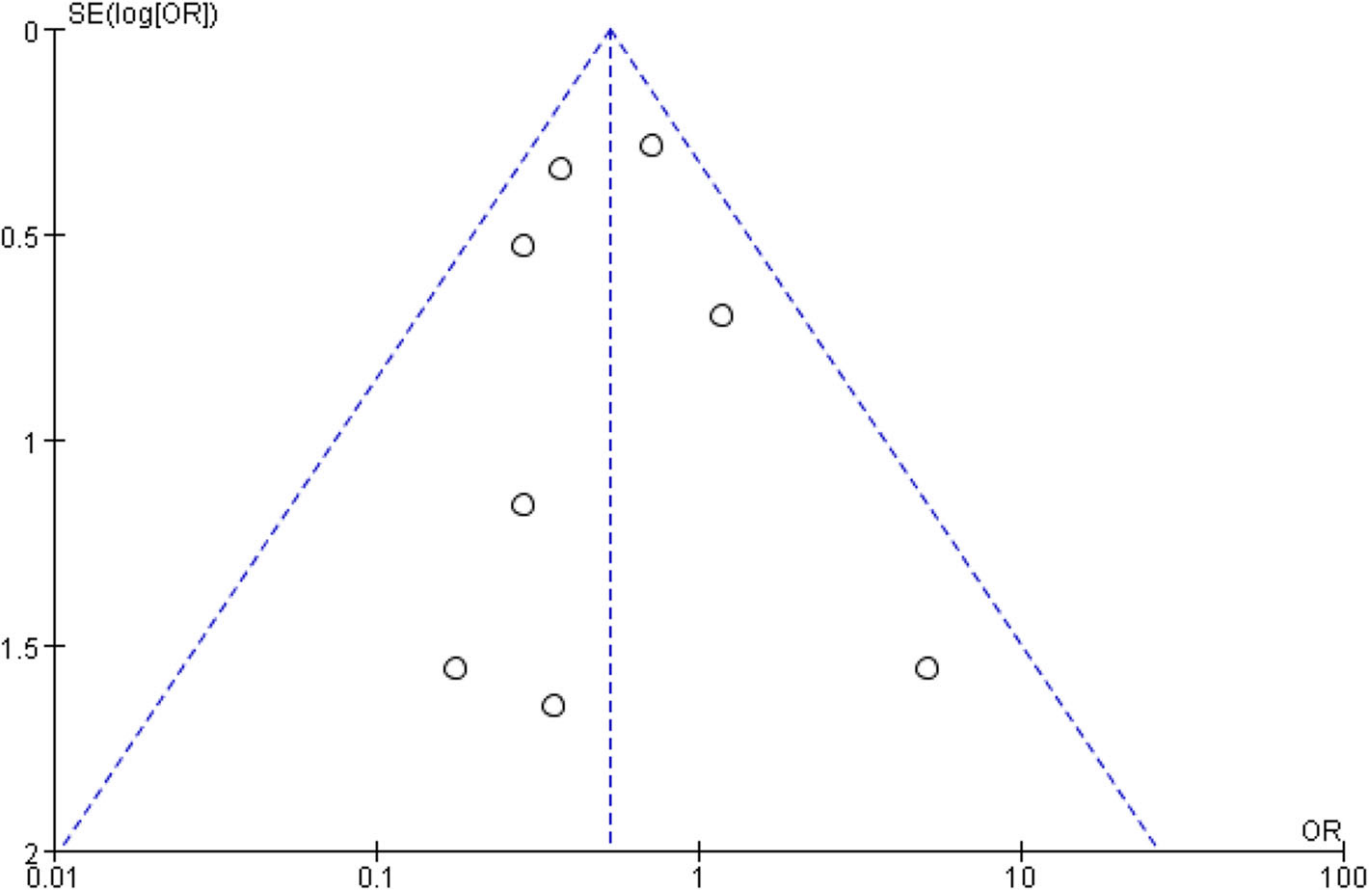
Funnel plot comparing of the intubation rate between high-flow nasal cannula oxygen (HFNC) and conventional oxygen therapy (COT) by Log odds ratio. OR odd ratio, SE standard error

Funnel plots were visually inspected and did not demonstrate evidence of publication bias (Fig. 4) The Egger regression test showed that the tests of asymmetry were not significant for any of the endpoints, including intubation rate for HFNC versus COT (OR −0.48, 95% CI −2.45, 1.65, *P* = 0.65); HFNC versus NIV (OR 0.27, 95% CI −331.57, 345.82, *P* = 0.83), and for secondary outcomes, HFNC versus COT for mechanical ventilation rate (OR −0.56, 95% CI −2.80, 1.72, *P* = 0.59), escalation rate (OR −1.73, 95% CI −3.35, 0.52, *P* = 0.13), and mortality rate (OR −0.28, 95% CI −3.17, 2.65, *P* = 0.80); HFNC versus COT for escalation rate (OR 0.60, 95% CI −125.28, 137.65, *P* = 0.66), and mortality rate (OR −0.75, 95% CI −203.51, 180.89, *P* = 0.80) (Additional file 4).

The GRADE quality evidence was assessed within GRADEpro software, and the results were as follows: for comparison of HFNC with COT, the quality of evidence on intubation rate and escalation rate was thought to be moderate, whereas evidence on the mechanical ventilation rate and mortality was thought to be low. The common reason for the demotion of RCTs was mainly a lack of blinding. For the rate of mechanical ventilation, the other reason was inconsistency, and for mortality, it was imprecision.

On assessment of the GRADE quality evidence of studies that compared HFNC with NIV, the quality of evidence on the intubation rate and mortality was thought to be very low, mainly because of the lack of blinding, inconsistency and imprecision. The quality of evidence on the escalation rate was thought to be low, and the main reason was a lack of blinding and imprecision. The summary of the findings and the quality of the analysis is provided in Table 2 and Table 3.

**Table 2:** Quality of evidence of the studies that compared HFNC to COT that were included in the meta-analysis, according to Grades of Recommendation, Assessment, Development and Evaluation (GRADE)

Outcomes	Anticipated absolute effects (95% CI)		Relative effect OR, (95% CI)	Participants (studies), *n*	Risk of bias	Inconsistency	Indirection	Imprecision	Publication bias	Quality of the evidence (GRADE)
	Risk with COT	Risk with HFNC								
Intubation rate	102/907 (11.2%)	67/947 (7.0%)	0.52 (0.34, 0.79)	1854 (8 RCTs)	Serious^a^	Not serious	Not serious	Not serious	Undetected	⨁⨁⨁◯ Moderate
Mechanical ventilation rate	145/937 (15.5%)	98/977 (10.0%)	0.56 (0.33, 0.97)	1914 (9 RCTs)	Serious^a^	Serious^b^	Not serious	Not serious	Undetected	⨁⨁◯◯ Low
Escalation rate	167/937 (17.8%)	98/977 (10.0%)	0.45 (0.31, 0.67)	1914 (9 RCTs)	Serious^a^	Not serious	Not serious	Not serious	Undetected	⨁⨁⨁◯ Moderate
Mortality	51/732 (6.8%)	57/765 (7.4%)	1.01 (0.67, 1.53)	1497 (5 RCTs)	Serious^a^	Not serious	Not serious	Serious^c^	Undetected	⨁⨁◯◯ Low

*COT* conventional oxygen therapy, *NIV* noninvasive mechanical ventilation, *HNFC* high-flow nasal cannula oxygen, *OR* odds ratio, *RCT* randomized controlled trial

^a^Unblinded intervention

^b^ *I* ^*2*^, 60%

^c^Wide CI

**Table 3:** Quality of evidence of studies that compared HFNC to NIV that were included in the meta-analysis, according to Grades of Recommendation, Assessment, Development and Evaluation (GRADE)

Outcomes	Anticipated absolute effects (95% CI)		Relative effect OR, (95% CI)	Participants (studies), *n*	Risk of bias	Inconsistency	Indirection	Imprecision	Publication bias	Quality of the evidence (GRADE)
	Risk with NIV	Risk with HFNC								
Intubation rate	172/841 (20.5%)	164/810 (20.9%)	0.96 (0.66, 1.39)	1651 (3 RCTs)	Serious^a^	Serious^b1^	Not serious	Serious^c^	Undetected	⨁◯◯◯ Very low
Escalation rate	206/841 (24.5%)	198/810 (24.4%)	1.00 (0.77, 1.28)	1651 (3 RCTs)	Serious^a^	Not serious	Not serious	Serious^c^	Undetected	⨁⨁◯◯ Low
Mortality	68/841 (8.1%)	59/810 (7.3%)	0.85 (0.43, 1.68)	1651 (3 RCTs)	Serious^a^	Serious^b2^	Not serious	Serious^c^	Undetected	⨁◯◯◯ Very low

*COT* conventional oxygen therapy, *NIV* noninvasive mechanical ventilation, *HNFC* high-flow nasal cannula oxygen, *OR* odds ratio, *RCT* randomized controlled trial

^a^Unblinded intervention

^b1^ *I* ^*2*^, 53%

^b2^ *I* ^*2*^, 69%

^c^Wide CI

### Primary outcomes

**Fig. 5:**
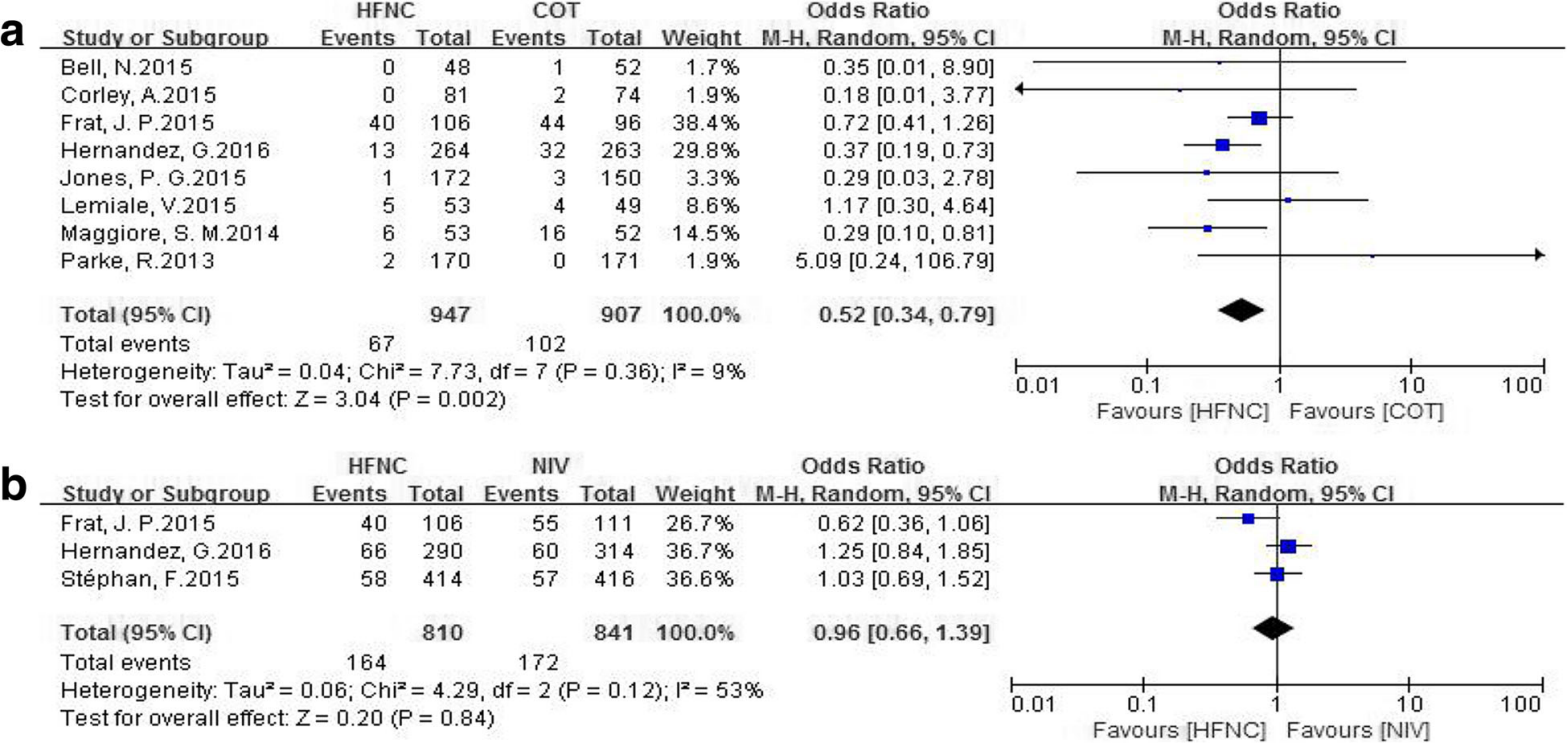
Comparison of intubation rates. **a** High-flow nasal cannula oxygen (*HFNC*) versus conventional oxygen therapy (*COT*). **b** HFNC versus noninvasive ventilation (*NIV*). *CI* confidence interval, *M-H* Mantel-Haenszel

Compared to COT, HFNC was associated with a significant reduction in the intubation rate (OR 0.52, 95% CI 0.34 to 0.79, *P* = 0.002; M-H random; *n* = 1854; heterogeneity *I* ^*2*^ = 9%, *P* = 0.36) (Fig. 5a).

No difference was found in the intubation rates between HFNC and NIV therapy (OR 0.96, 95% CI 0.66 to 1.39, *P* = 0.84; M-H random; *n* = 1651; heterogeneity *I* ^*2*^ = 53%, *P* = 0.12) (Fig. 5b).

### Secondary outcomes

**Fig. 6:**
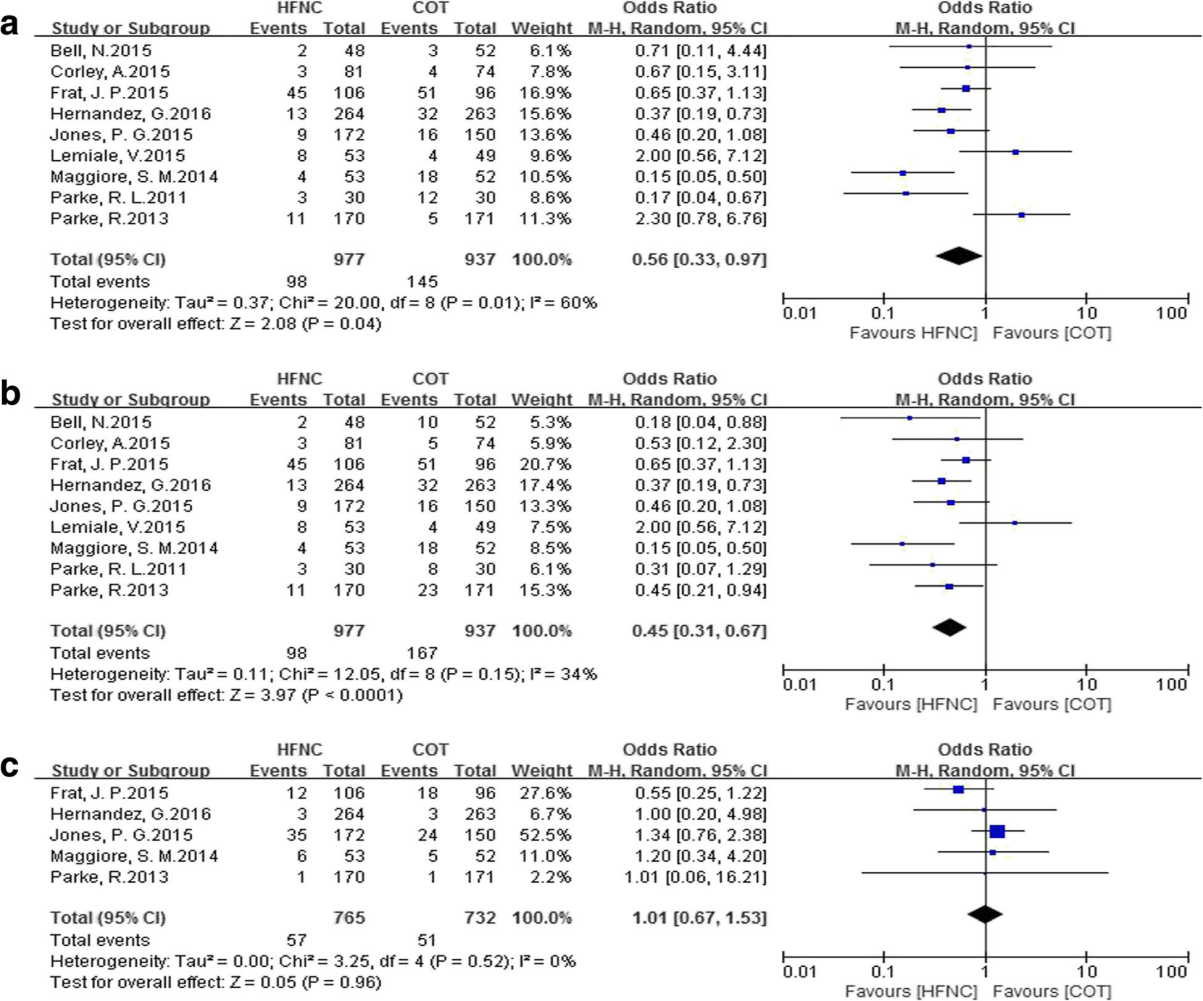
Comparison of secondary outcomes in patients who received high-flow nasal cannula oxygen (*HFNC*) compared to conventional oxygen therapy (*COT*). **a** Effect on the rate of mechanical ventilation. **b** Effect on the rate of escalation of respiratory support. **c** Effect on mortality. *CI* confidence interval, *M-H* Mantel-Haenszel

Nine RCTs that recruited 1914 patients showed that the use of HFNC significantly reduced the mechanical ventilation rate (OR 0.56, 95% CI 0.33 to 0.97, *P* = 0.04), and the heterogeneity was moderate with *I* ^*2*^ = 60% for heterogeneity (*P* = 0.01, M-H random) (Fig. 6a).

The overall rate of escalation of respiratory support was also significantly lower in the HFNC group when compared with the COT group (OR 0.45, 95% CI 0.31 to 0.67, *P* < 0.00001), and the heterogeneity was low with *I* ^*2*^ = 34% for heterogeneity (*P* = 0.15, M-H random) (Fig. 6b). Only five RCTs [18–22] expressed data on mortality, and there was no difference between HFNC and COT therapies (OR 1.01, 95% CI 0.67 to 1.53, *P* = 0.96; M-H random; *n* = 1497; heterogeneity *I* ^*2*^ = 0%, *P* = 0.52) (Fig. 6c).

**Fig. 7:**
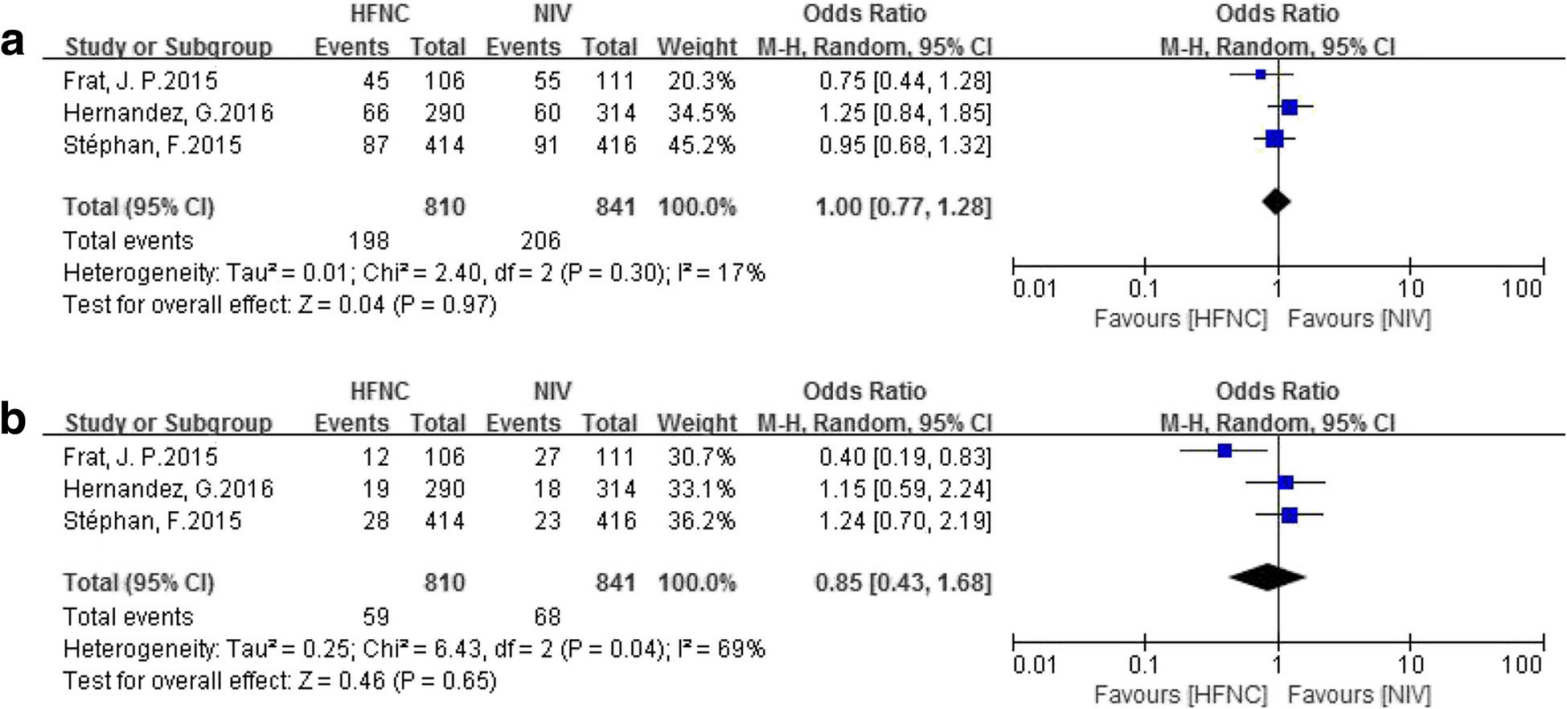
Comparison of secondary outcomes in patients who received high-flow nasal cannula oxygen (*HFNC*) compared to noninvasive ventilation (*NIV*). **a** Effect on the rate of escalation of respiratory support. **b** Effect on mortality. *CI* confidence interval, *M-H* Mantel-Haenszel

Three RCTs that included 1651 patients compared HFNC with NIV. There was no significant difference in the rate of escalation of respiratory support (OR 1.00, 95% CI 0.77 to 1.28, *P* = 0.97; M-H random; heterogeneity *I* ^*2*^ = 17%, *P* = 0.30) (Fig. 7a) or in mortality (OR 0.85, 95% CI 0.43 to 1.68, *P* = 0.65; M-H random; heterogeneity *I* ^*2*^ = 69%, *P* = 0.04) (Fig. 7b).

### Subgroup analysis of HFNC versus COT

#### Reasons for ARF

Subgroup analysis showed that four trials included patients with post-extubation ARF [19, 21, 22, 44] and were associated with a significant reduction in the intubation rate (OR 0.37, 95% CI 0.20 to 0.70, *P* = 0.002; M-H random; *n* = 1128; heterogeneity *I* ^*2*^ = 10%, *P* = 0.34). However, the other four trials included patients with AFR due to other reasons [13, 18, 20, 45], and there was no significant effect on the intubation rate (OR 0.72, 95% CI 0.44 to 1.19, *P* = 0.20; M-H random; *n* = 726; heterogeneity *I* ^*2*^ = 0%, *P* = 0.73).

#### Escalation stage

In the subgroup analysis, a significant reduction was also observed (OR 0.51, 95% CI 0.32 to 0.81, *P* = 0.004; M-H random; *n* = 1258; heterogeneity *I* ^*2*^ = 21%, *P* = 0.28) when COT was not allowed to progress to HFNC [18–21, 45]. However, no difference was observed (OR 0.70, 95% CI 0.09 to 5.37, *P* = 0.73; M-H random; *n* = 596; heterogeneity *I* ^*2*^ = 23%, *P* = 0.27) when COT was allowed to progress to HFNC [13, 22, 44].

#### Type of study design

In the subgroup analyses of the two single-centre trials [20, 22], there was no effect on the intubation rate (OR 1.01, 95% CI 0.06 to 16.73, *P* = 1.00; M-H random; *n* = 663; heterogeneity *I* ^*2*^ = 55%, *P* = 0.14). However, in six multi-centre trials [13, 18, 19, 21, 44, 45], there was a significant reduction in the incidence of intubation (OR 0.51, 95% CI 0.34 to 0.77, *P* = 0.001; M-H random; *n* = 1191; heterogeneity *I* ^*2*^ = 6%, *P* = 0.38). The primary and secondary outcomes of the subgroup analysis are shown in Additional file 5.

## Discussion

The main finding of our study was that HFNC significantly reduced the rate of intubation, mechanical ventilation and escalation of respiratory support compared with COT in adult patients with respiratory failure, but there was no difference in mortality. On the other hand, when compared to NIV, no significant difference in intubation rate, escalation of respiratory support rate or mortality was detected.

Our results were partially different from two recent meta-analyses of HFNC in adult patients [26, 27]. Our meta-analysis was registered on the PROSPERO website before the other authors published, and we included more RCTs (11 RCTs recruiting 3459 patients); Maitra et al. only included 6 RCTs in their meta-analysis and only compared the prognosis of higher respiratory support, while also finding no significant difference between HFNC and standard oxygen therapy or NIV [27]. We included more outcomes for HFNC, including intubation rate, mechanical ventilation rate, the rate of escalation of respiratory support and mortality. Furthermore, we focused on various prognostic indicators of HFNC compared with COT or NIV, to decrease the population heterogeneity and population bias of the sample.

Our meta-analysis found that HFNC also significantly reduced the rate of intubation, mechanical ventilation and the escalation of respiratory support compared to COT. HFNC is superior to COT due to the delivery of heated humidified oxygen, higher and more predictable gas flow rates and rates of FiO_2_, a positive pressure effect and improvement in sputum clearance [3, 4]. Nevertheless, HFNC did not reduce mortality when compared to COT, which could be explained by the complex causes of respiratory failure. Frat et al. [18] enrolled patients in the ICU with acute respiratory failure, most of which had community-acquired pneumonia. Jones et al. [20] included patients in the ED with respiratory failure with the main cause indicated as chronic obstructive pulmonary disease (COPD). The other three RCTs included patients with respiratory failure post extubation [19, 21, 22], and patients in the study of Parke et al. [22] were included after undergoing cardiac surgery. In addition, in three RCTs [13, 22, 44] COT was allowed to escalate to HFNC, and the subgroup analysis found no significant differences in the intubation rate between the two groups, which suggests early conversion to HFNC after failure of COT. Care should also be taken since some patients could not tolerate HFNC: in the study of Jones [20], 11 of 172 patients did not tolerate HFNC and were placed on COT. Finally, the prolonged use of HFNC could result in delayed intubation and increased mortality [48].

NIV has been proven to improve gas exchange and reduce the need for intubation and mortality in patients with respiratory failure, especially in patients with an exacerbation of COPD and those with acute cardiogenic pulmonary oedema [49, 50]. Compared with NIV, HFNC has some advantages, such as greater patient comfort, lower costs, increased ease in clearing secretions [34] and a lower incidence of adverse effects that could lead to a poorer outcome [18]. We included three RCTs that showed no difference between HFNC and NIV in terms of intubation rate and mortality, which suggests that HFNC was not inferior to NIV pertaining to the patients’ prognoses, at least in patients with hypoxaemic respiratory failure. Of the three RCTs, Frat et al. [18] enrolled patients in the ICU with acute hypoxaemic respiratory failure and excluded chronic respiratory failure and cardiogenic pulmonary oedema. A study by Stephan [47] enrolled hypoxemic patients after cardiothoracic surgery. Hernandez et al. [25] included patients with post-extubation respiratory failure and excluded those with hypercapnia. The exacerbation of COPD and acute cardiogenic pulmonary oedema may require further study.

A few RCTs [19, 22, 25] showed that the length of ICU stay was not influenced by choice. Parke et al. [22] reported no difference in HFNC compared with COT (HFNC 33.4 h versus COT 28.9 h, *P* = 0.08 [22], and Hernandez et al. [19, 25] reported that the patients’ length of stay in the ICU was similar between HFNC and COT (6 days (IQR 2–8) with HFNC versus 6 days (IQR 2–9) with COT, *P* = 0.29), and between HFNC and NIV (9 days (IQR 4–19) in HFNC versus 10.5 days (IQR 5–19) in NIV, *P* = 0.23). Thus, more RCTs are necessary in the future.

There are several limitations to our meta-analysis. First, the causes of the respiratory distress were heterogeneous in the patients recruited for the studies included. The studies included in the meta-analysis had been performed by researchers who operated independently, and the subjects or interventions in these studies would have differed in ways that could have impacted the results; therefore, we used a random-effects model to make the outcomes conservative and easy to justify. The subgroup analysis showed that HFNC may be more suitable for patients with post-extubation ARF. Second, the duration of treatment and the modes of HFNC and NIV were also variable, which further increased the heterogeneity. Third, all the RCTs included had a high risk of performance bias due to the dramatic differences between HFNC, COT and NIV, which made blinding impossible. Finally, all the studies included in this meta-analysis received sponsorship from Fisher and Paykel Healthcare, although most of the articles [13, 18–20, 44–46] state that the study design and data analysis were independent of commercial interference.

## Conclusions

In conclusion, compared with COT, HFNC reduced the rate of intubation, mechanical ventilation and escalation of respiratory support. When compared to NIV, HFNC showed similar results. Large-scale randomized controlled trials are necessary to confirm our findings.

## Key messages

- HFNC showed promise in reducing the need for intubation, mechanical ventilation and escalation of respiratory support compared to COT. Large-scale randomized controlled trials are needed to provide more robust evidence.

## Additional files

Additional file 1: Search strategy. (DOCX 16 kb)

Additional file 2: Table of studies excluded from the meta-analysis. (DOCX 19 kb)

Additional file 3: Table of quality assessment of the included RCTs using the Cochrane Collaboration tool. (DOCX 17 kb)

Additional file 4: Egger’s regression figures for primary or secondary outcomes of HFNC versus COT. **a** Intubaiton rate. **b** Mechanical ventilation rate. **c** Escalation rate. **d** Mortality. (PDF 433 kb)

Additional file 5: Table of subgroup analysis of HFNC versus COT. (DOCX 18 kb)

## Abbreviations

AHRF: Acute hypoxaemic respiratory failure
ARF: Acute respiration failure
BMI: Body mass index
CI: Confidence interval
COPD: Chronic obstructive pulmonary disease
COT: Conventional oxygen therapy
CTVS: Cardiothoracic and vascular surgery
ED: Emergency department
FiO_2_: Fraction of inspired oxygen
FM: Face mask
GRADE: Grading of recommendations assessment, development, and evaluation
HFNC: High-flow nasal cannula oxygen
ICU: Intensive care unit
MD: Mean difference
M-H: Mantel-Haenszel
NIV: Non-invasive ventilation
OR: Odd ratio
PD: Pneumology department
PRISMA: Preferred Reporting Items for Systematic Reviews and Meta-Analyses
RCT: Randomized controlled trial
RF: Respiratory failure
SE: Standard error
