# Supplementary material for: High-flow nasal cannula oxygen therapy is superior to conventional oxygen therapy but not to noninvasive mechanical ventilation on intubation rate: a systematic review and meta-analysis
Source: Crit Care. 2017 Jul 12;21:184. doi: 10.1186/s13054-017-1760-8 (PMC5508784; doi:10.1186/s13054-017-1760-8)
Supplement: Supplementary file 1 — Search strategy. (DOCX 16 kb) [file 13054_2017_1760_MOESM1_ESM.docx]

**Additional file 1：**

**APPENDIX:**

**Appendix 1. CENTRAL search strategy**

1. ((high frequency or high flow) NEAR/5 nasal):ti,ab,kw (Word variations have been searched)

2. ((high frequency or high flow) NEAR/5 oxygen):ti,ab,kw (Word variations have been searched)

3. ( high-flow NEAR/5 nasal):ti,ab,kw (Word variations have been searched)

4. (nasal NEAR/5 (high flow)):ti,ab,kw (Word variations have been searched)

5. (hfnc or hfnp or hhfnox or hfno):ti,ab,kw (Word variations have been searched)

6 .1 or 2 or 3 or 4 or 5

**Appendix 2. MEDLINE ( Ovid SP ) search strategy**

1.((high frequency or high flow) adj5 nasal).tw.

2. ((high frequency or high flow) adj5 oxygen).tw.

3 .( high-flow adj5 nasal).tw.

4. (nasal adj5 (high flow)).tw.

5. (hfnc or hfnp or hhfnox or hfno).tw.

6 .1 or 2 or 3 or 4 or 5

7. limit 6 to ("all adult (19 plus years)" and (chinese or english))

Search studies published up to October 20, 2016

**Appendix3. Embase.com search strategy**

#7#6([chinese]/lim or [english]/lim] and ([adult]/lim or [aged]/lim)

#6 #1 OR #2 OR #3 OR #4 OR #5

#5 hfnc:ab,ti OR hfnp:ab,ti OR hhfnox:ab,ti OR hfno:ab,ti

#4 (nasal NEAR/5 (‘high flow’ )):ab,ti

#3 (( ‘high-flow’ ) NEAR/5 nasal):ab,ti

#2 (( ‘high frequency’ OR ‘high flow’ ) NEAR/5 oxygen):ab,ti

#1 (( ‘high frequency’ OR ‘high flow’ ) NEAR/5 nasal):ab,ti

Search studies published up to October 20, 2016
