# Supplementary material for: High-flow nasal cannula oxygen therapy is superior to conventional oxygen therapy but not to noninvasive mechanical ventilation on intubation rate: a systematic review and meta-analysis
Source: Crit Care. 2017 Jul 12;21:184. doi: 10.1186/s13054-017-1760-8 (PMC5508784; doi:10.1186/s13054-017-1760-8)
Supplement: Supplementary file 2 — Table of studies excluded from the meta-analysis. (DOCX 19 kb) [file 13054_2017_1760_MOESM2_ESM.docx]

**Additional file 2: Table of studies excluded from the meta-analysis**

| Study | Setting | Design | N | Population | Control | Report intubation | Report mortality | Reason to exclusion |
| --- | --- | --- | --- | --- | --- | --- | --- | --- |
| Brotfain E.2014 | ICU | [Retrospectivestudy](file:///C:\%25E8%25BD%25AF%25E4%25BB%25B6\Youdao\Dict\6.3.69.8341\resultui\frame\javascript:void(0);) | 67 | Post-extubation | COT | NO | YES | NOT RCT |
| Cuquemelle E.2015 | ICU | Crossover | 30 | AHRF | COT | YES | NO | NOT RCT |
| Frat JP.2015 | ICU | Observational study | 28 | AHRF | NIV | YES | NO | NOT RCT |
| Rittayamai N.2014 | ICU | Crossover | 17 | Post-extubation | COT | YES | NO | NOT RCT |
| Roca O.2015 | ICU | Retrospective  cohort analysis | 37 | ARF | COT | YES | NO | NOT RCT |
| Sztrymf B.2012 | ICU | Observationa study | 20 | ARF | COT | YES | YES | NOT RCT |
| Tiruvoipati R.2010 | ICU | Crossover | 50 | Post-extubation | COT | YES | YES | NOT RCT |
| Yoo JW.2016 | ICU | Retrospective cohort analysis | 73 | Post-extubation | NIV | YES | YES | NOT RCT |
| Hui D.2013 | ICU | Crossover | 30 | Persistent dyspnea(with cancer ) | NIV | NO | NO | Not include our outcomes |
| Lucangelo U.2012 | PD | RCT | 45 | Pulmonary disease undergoing fibreoptic bronchoscopy | COT | NO | NO | Not include our outcomes |
| Rittayamai N.2015 | ED | RCT | 40 | Acute dyspnea and hypoxemia | COT | NO | NO | Not include our outcomes |
| Schwabbauer N.2014 | ICU | Crossover | 14 | ARF | COT/ NIV | NO | NO | Not include our outcomes |
| Simon M.2014 | ICU | RCT | 40 | RF undergoing bronchoscopy | NIV | YES | YES | Applying HFNC for bronchoscopy, not for treatment |
| Futier E.2013 | ICU | RCT | 220 | Post-extubatioon | COT | --- | --- | Without complete information |
| Yildirim F.2015 | ICU | --- | --- | Respiratory Distress Syndrome. | NIV | --- | --- | Not English/Chinese language |

ICU, Intensive care units; COT, Conventional oxygen therapy; NIV, Noninvasive mechanical ventilation; ED, Emergency department; AHRF, Acute hypoxemic respiratory failure; AFR, Acute respiratory failure; FR, Respiratory failure; PD, Pneumology department
