# Supplementary material for: High-flow nasal cannula oxygen therapy is superior to conventional oxygen therapy but not to noninvasive mechanical ventilation on intubation rate: a systematic review and meta-analysis
Source: Crit Care. 2017 Jul 12;21:184. doi: 10.1186/s13054-017-1760-8 (PMC5508784; doi:10.1186/s13054-017-1760-8)
Supplement: Supplementary file 3 — Table of quality assessment of the included RCTs using the Cochrane Collaboration tool. (DOCX 17 kb) [file 13054_2017_1760_MOESM3_ESM.docx]

**Additional file 3：Quality assessment of RCTs included by the Cochrane Collaboration tool**

| Study | Selection bias | | Performance bias  Blinding of participants and personnel | Detection bias  Blinding of outcome assessment | Attrition bias  Incomplete outcome data assessments | Reporting bias  Selective reporting | Other bias  Receive sponsorship or Oxygen therapy devices |
| --- | --- | --- | --- | --- | --- | --- | --- |
|  | Random sequence generation | Allocation concealment |  |  |  |  |  |
| Bell N.2015 | Low | Low | High | Low | Low | Low | Low |
| Corley A.2015 | Low | Low | High | Low | Low | Low | Low |
| Frat JP.2015 | Low | Low | High | Low | Low | Low | Low |
| Hernandez G.2016 | Low | Low | High | Low | Low | Low | Low |
| JonesPG. 2015 | Low | Low | High | Low | Low | Low | Low |
| Lemiale V.2015 | Unclear | Low | High | Low | Low | Low | Low |
| Maggiore,SM. 2014 | Low | Low | High | Low | Low | Low | Unclear |
| Parke R.2013 | Low | Low | High | Low | Low | Low | Unclear |
| Parke R.L. 2011 | Low | Low | High | Low | Low | Low | Low |
| Stéphan, F. 2015 | Unclear | Low | High | Low | Low | Low | Unclear |

Low, low risk of bias; High, high risk of bias; Unclear, unclear risk of bias according to the relative information
