# Supplementary figures and images for: High-flow nasal cannula oxygen therapy is superior to conventional oxygen therapy but not to noninvasive mechanical ventilation on intubation rate: a systematic review and meta-analysis
Source: Crit Care. 2017 Jul 12;21:184. doi: 10.1186/s13054-017-1760-8 (PMC5508784; doi:10.1186/s13054-017-1760-8)

(a)

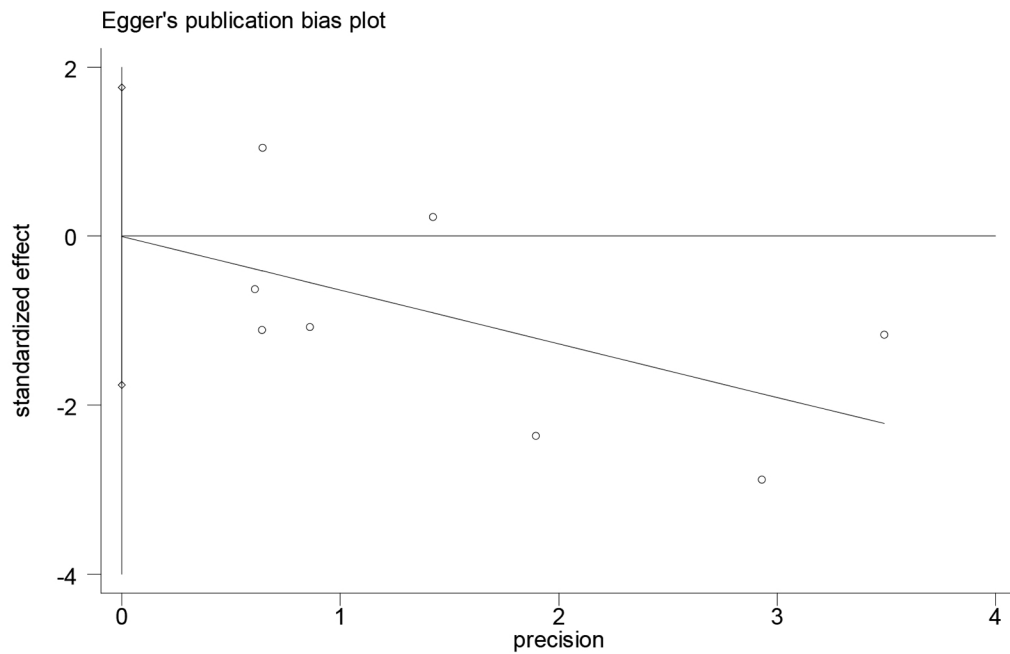

(b)

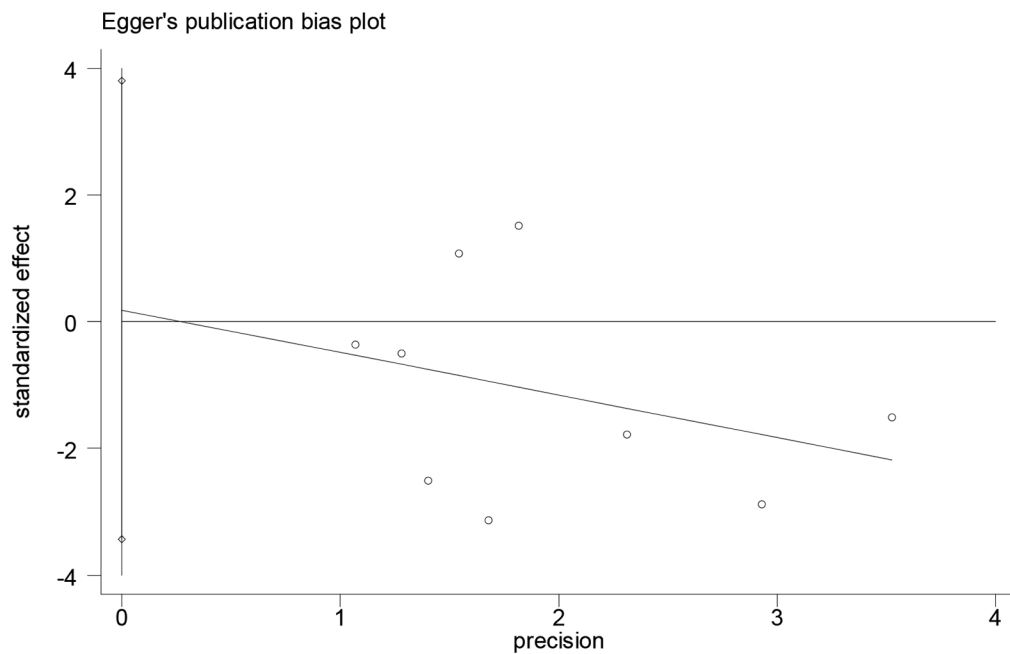

(c)

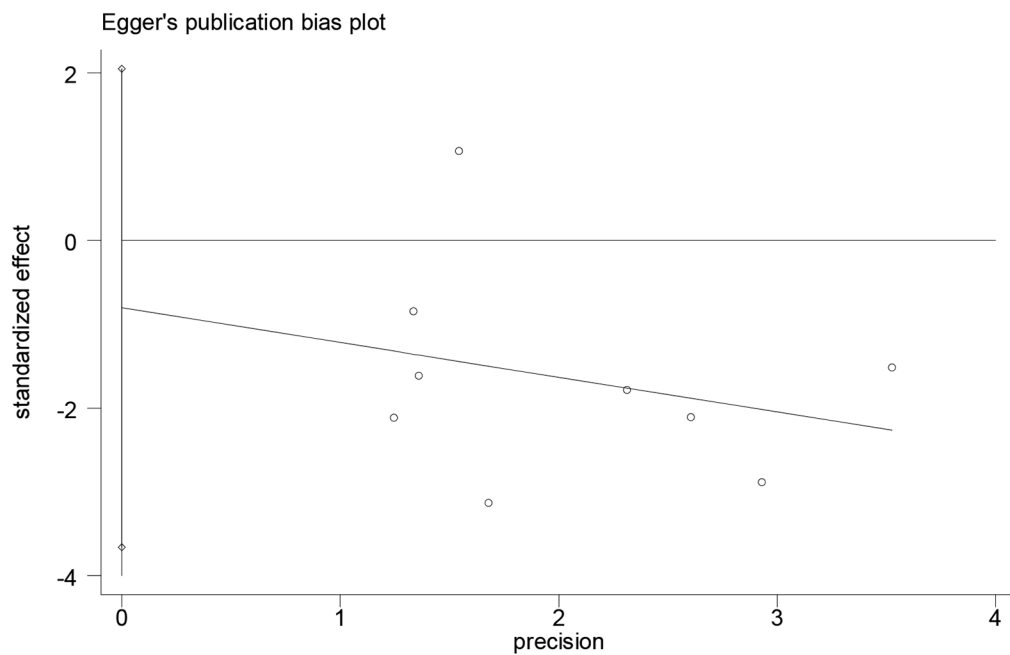

(d)

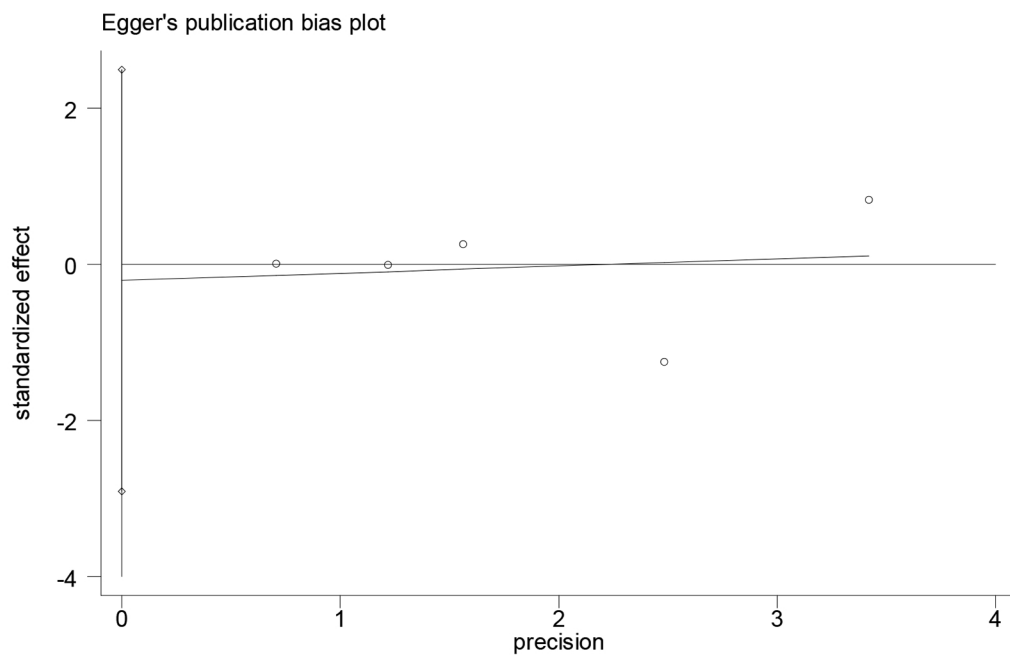

Supplement: Supplementary file 4 — Egger’s regression figures for primary or secondary outcomes of HFNC versus COT. a Intubaiton rate. b Mechanical ventilation rate. c Escalation rate. d Mortality. (PDF 433 kb) [file 13054_2017_1760_MOESM4_ESM.pdf]
