# Supplementary material for: High-flow nasal cannula oxygen therapy is superior to conventional oxygen therapy but not to noninvasive mechanical ventilation on intubation rate: a systematic review and meta-analysis
Source: Crit Care. 2017 Jul 12;21:184. doi: 10.1186/s13054-017-1760-8 (PMC5508784; doi:10.1186/s13054-017-1760-8)
Supplement: Supplementary file 5 — Table of subgroup analysis of HFNC versus COT. (DOCX 18 kb) [file 13054_2017_1760_MOESM5_ESM.docx]

**Table 6. Subgroup analysis of HFNC vs. COT**

|  | No.of studies | HFNC  Event/total | COT  Event/total | *I^2^*(%) | *P* value for  heterogeneity | OR 95%CI | Overall effect  Z/P value |
| --- | --- | --- | --- | --- | --- | --- | --- |
|  |  |  |  |  |  |  |  |
| Intubation rate |  |  |  |  |  |  |  |
| Reasons of ARF |  |  |  |  |  |  |  |
| Postextubation | 4 | 21/568 | 50/560 | 10 | 0.34 | 0.37 [0.20, 0.70] | 3.09/0.002 |
| Other reasons | 4 | 46/379 | 52/347 | 0 | 0.73 | 0.72[0.44, 1.19] | 1.29/0.20 |
| Escalation stage |  |  |  |  |  |  |  |
| Allow COT escalate to HFNC | 3 | 2/299 | 3/297 | 23 | 0.27 | 0.70[0.09,5.37] | 0.34/0.73 |
| COT not escalate to HFNC | 5 | 65/648 | 99/610 | 21 | 0.28 | 0.51[0.32,0.87] | 2.88/0.04 |
| Type of study design |  |  |  |  |  |  |  |
| Single-center | 2 | 3/342 | 3/321 | 55 | 0.14 | 1.01[0.06,16.73] | 0.00/1.00 |
| Multi-center | 6 | 64/605 | 99/586 | 6 | 0.38 | 0.51[0.34,0.77] | 3.23/0.001 |
| Mechanical ventilation |  |  |  |  |  |  |  |
| Reasons of ARF |  |  |  |  |  |  |  |
| Postextubation | 4 | 31/568 | 59/560 | 76 | 0.006 | 0.54[0.18,1.59] | 1.12/0.26 |
| Other reasons | 5 | 67/409 | 86/377 | 44 | 0.13 | 0.59[0.32,1.11] | 1.63/0.10 |
| Escalation stage |  |  |  |  |  |  |  |
| Allow COT escalate to HFNC | 3 | 16/299 | 12/297 | 9 | 0.33 | 1.29[0.55,3.00] | 0.59/0.56 |
| COT not escalate to HFNC | 6 | 82/678 | 133/640 | 60 | 0.03 | 0.44[0.25,0.79] | 2.76/0.006 |
| Type of study design |  |  |  |  |  |  |  |
| Single-center | 3 | 23/372 | 33/351 | 79 | 0.009 | 0.59[0.15,2.32] | 0.76/0.45 |
| Multi-center | 6 | 75/605 | 112/586 | 51 | 0.07 | 0.54[0.30,0.98] | 2.04/0.04 |
| Escalation rate |  |  |  |  |  |  |  |
| Reasons of ARF |  |  |  |  |  |  |  |
| Postextubation | 4 | 31/568 | 78/560 | 0 | 0.45 | 0.36[0.23,0.56] | 4.55/0 |
| Other reasons | 5 | 67/409 | 89/377 | 42 | 0.14 | 0.56[0.31,1.02] | 1.89/0.06 |
| Escalation stage |  |  |  |  |  |  |  |
| Allow COT escalate to HFNC | 3 | 16/299 | 38/297 | 0 | 0.55 | 0.40[0.22,0.74] | 2.91/0.004 |
| COT not escalate to HFNC | 6 | 82/678 | 129/640 | 53 | 0.06 | 0.47[0.28,0.81] | 2.73/0.006 |
| Type of study design |  |  |  |  |  |  |  |
| Single-center | 3 | 23/372 | 47/351 | 0 | 0.88 | 0.43[0.25,0.73] | 3.16/0.002 |
| Multi-center | 6 | 75/605 | 120/586 | 57 | 0.04 | 0.46[0.25,0.86] | 2.45/0.01 |
| Mortality |  |  |  |  |  |  |  |
| Reasons of ARF |  |  |  |  |  |  |  |
| Postextubation | 3 | 10/478 | 9/486 | 0 | 0.98 | 1.11[0.44,2.81] | 0.21/0.83 |
| Other reasons | 2 | 47/278 | 42/246 | 68 | 0.08 | 0.90[0.38,2.13] | 0.24/0.81 |
| Escalation stage |  |  |  |  |  |  |  |
| Allow COT escalate to HFNC | 1 | 1/170 | 1/171 | NA | NA | 1.01[0.06,16.21] | 0.00/1.00 |
| COT not escalate to HFNC | 4 | 56/595 | 50/561 | 8 | 0.36 | 1.00[0.64,1.57] | 0.01/0.99 |
| Type of study design |  |  |  |  |  |  |  |
| Single-center | 2 | 36/342 | 25/321 | 0 | 0.84 | 1.33[0.76,2.32] | 0.98/0.32 |
| Multi-center | 3 | 21/423 | 26/411 | 0 | 0.54 | 0.73[0.39,1.35] | 1.01,0.31 |

HFNC, High flow nasal cannula; COT, Conventional oxygen therapy; OR, Odds ratio;

CI, Confidence interval; ARF, Acute respiratory failure
